# Mr. Ring, on Vaccination

**Published:** 1807-03

**Authors:** John Ring

**Affiliations:** New-street, Hanover-square


### To the Editors of the Medical and Phyſical Journal.

Gentlemen,

In the Medical and Chirurgical Review, for January, is an account of the case of Mr. Dawes's child, communicated to the Vaccine Club by Mr. Millington. This patient, it is truly stated, was vaccinated by me seven years ago. It is also truly stated, that she was thought safe from the small-pox; and there was reason to think her as safe as any kind of inoculation could make her; since she was afterwards inoculated in both arms with fresh and active variolous matter, and repeatedly exposed to the infection of the natural small-pox, which she resisted.

I saw her on the fifth day of the eruption, which lately appeared in consequence of her exposure to the variolous infection, when sleeping with her brother, who was inoculated for the small-pox by Mr. Millington; and had no doubt that the eruptions were occasioned thereby; but was doubtful whether they were occasioned by effluvia, or by contact. It appeared to me probable, that those which contained a fluid were occasioned by the latter. Indeed, scarcely any appeared to contain a fluid, except one on the wrist; which was semi-pellucid. The rest were turned brown, though it was only the fifth day.

It is, perhaps, of but little moment, whether the pustules are produced by contact or effluvia, unless in this respect, that if they arise from contact, they may be the more easily avoided in other cases.

Mr. Millington says, I hesitated to acknowledge the eruption to be the small-pox. This is rather inconsistent with Mr. Dawes's account; for in a letter to the Royal College of Physicians, he says, I admitted it to be that disorder. Mr. Millington was not present at the time; and cannot be supposed to be a competent evidence; but he informs me, that he drew this conclusion from the account given to him by Mrs. Bailey, a relation of Mr. Dawes; who was present when I saw the case.

He says, Mrs. Bailey told him, that I admitted it to be the small-pox, in the same manner in which a person may have it, who has already had the disease. This is the real truth; but Mr. Millington confesses he did not know, that a person who has already had the small-pox, may have *local pustules capable of communicating infection to others*

*by inoculation.* That fact, however, is well established; and now generally known.

Two circumstances are proper to be mentioned relative to this case. The first is, that this patient was vaccinated in the very infancy of the practice, with matter obtained from Dr. Pearson; who took matter from the vesicle of this patient, by drawing a string backwards and forwards through it, which occasioned a sore arm. This was done, when the disorder was on *the decline*; a proof, that he was then in the habit of taking matter *at a late period.* Whether, therefore, the matter with which this patient was inoculated was less active, or the security diminished by the injury done to the vesicle, may be a question. The cicatrix is large and irregular; and seems to bear marks of the violence which the vesicle sustained. Matter taken from the variolous pustule on the wrist of this child, by Mr. Robinson, of Duke-street, Grosvenor-square, proved successful in inoculation; and excited the small-pox.

While the supposed failures of vaccination are brought forward, those of variolation ought also to be stated. One case of this kind was related to me by Mrs. Bailey, when I called on her to make the inquiry concerning Miss Dawes. It occurred in a young woman at Reading, with whom Mrs. Bailey was well acquainted. She had been inoculated for the small pox, and was supposed to be safe; but she afterwards caught the natural disorder, not in an equivocal manner, like Miss Dawes; but in such a degree, that it proved fatal.

Another case of the same sort was communicated to me by Mr. Millington himself, when returning from the Royal College of Physicians, where we had just been stating the particulars of Miss Dawes's case, to which this may be considered as much more than a counterpoise. The subject of it was a child of Mr. Locke, No. 5, Little Castle-street, Oxford Market, now six years of age. She was inoculated for the small-pox by Mr. Millington, when about a year old. Her arm rose very favourably. She had no eruption. The pustule was broken by some accident, about the ninth day.

Three years after, she was exposed to the infection of the small-pox; in consequence of which she caught the disorder; and, according to the account of her parents, had about five or six hundred pustules. They maturated, and were very confluent on one cheek. Mr. Millington attended her; and he, as well as her parents, was perfectly convinced that it was the small-pox.

Mr.

Mr. Locke informs me, that in another child in his house, inoculated for the small-pox, the arm did not begin to inflame till the 40th day. This circumstance, though totally foreign to the former subject, appeared to me worthy of being recorded. The only instance like it, that has fallen under my observation, or come to my knowledge, is that of the child of Mr. Dust, a gun-maker of Chelsea, vaccinated by me, whose arm did not begin to inflame till the 46th day. In that case, already published in this Journal, the progress of vaccination was probably retarded by the counter-stimulus of a very large tetter.

In the case of Mr. Locke's child, Mr. Millington, who inoculated her, was perfectly satisfied with the operation, and on that account did not inoculate her again. He was astonished when he saw the disorder appear afterwards in the natural way. Whether this failure was owing to the injury done to the pustule, is not easy to be determined.

It is of little consequence to the patient who suffers an unexpected attack of a disorder, whether the failure is owing to the practitioner or the practice; but when gentlemen are so ready to proclaim, and Vaccine Clubs, or Anti-vaccine Societies, to publish the failures of the cow-pock, and not those of the small-pox, there is something ambiguous in their conduct. It is natural to suspect, that they wish to bring vaccination into disrepute, in order to revive the inoculation of the small-pox. To use the expression of the late head of the Anti-vaccinists, *there is something rotten in the state of Denmark.*

To the same cause we are to attribute the many unfounded insinuations which are met with, concerning the friends of vaccination. The enemies of the practice, not satisfied with asserting that the small-pox occurs after the cow-pock, artfully pretend, that the friends of the practice are unwilling to acknowledge the fact.

To the comunication of Mr. Millington, in the Medical and Chirurgical Review, are added some invidious and illiberal observations, worthy of that man, who has endeavoured to rob Dr. Jenner of his merited honours; and, if we may use Mr. Moore's expression, *to pilfer his laurel crown.* This gentleman, who is so quick-sighted in discovering the failures in vaccination, can discover no failures in the small-pox. He has the modesty to maintain, that, at least, fifty times as many persons have the small-pox after the cow-pock, as were *ever pretended to have had it after variolous inoculation.*

 This

This is so gross and palpable a falsehood, that it cannot be suffered to pass unrefuted. The Edinburgh Reviewers, in the last number of their work, published October 16th, 1806, say;

"*It is the opinion of Dr. Willan, and seems to be confirmed by the import of the whole evidence, that vaccine inoculation bestows as great security, at least, as that for the small-pox. We think it has been demonstrated beyond the possibility of contradiction, that the number of authenticated cases of small-pox after the old inoculation, and even after a former attack of the natural disease, is greater in proportion, than those which are alleged, with any probability, of such an occurrence after complete vaccination.*"

The author of the remarks in the Medical and Chirurgical Review says, it appears in history, that "the opposition to variolous inoculation, for some years after its introduction, *was not so much on the ground of insecurity from the practice, as is at present the case with regard to vaccine inoculation.*" This shews either a total ignorance of the history of variolous inoculation, or the most unblushing effrontery. In my answer to Dr. Moseley, I observed, that Sir Richard Blackmore endeavoured to prove, on the authority of Mr. Tanner, a surgeon of St. Thomas's Hospital, *that variolous inoculation affords no security against the small-pox*; and that he did not rest his argument on Mr. Tanner's case alone; but spoke of it as *by no means singular.* "He thought *the idea of inoculation proving a preservative against the natural small-pox, contrary to experience and observation.*" He relates one case of failure, which occurred in the practice of Mr. Maitland.

The Rev. Mr. Massey, in his sermon preached at St. Andrews, affirms, that "*the confessed miscarriages in this new method, are more than happen in the ordinary way*; that inoculators are *physicians of no value*, and *forgers of lies, who confidently tell us what it is impossible for them to know, that those persons who are inoculated, are thereby for ever secured from any future danger of infection.*" He says, *this is a bold assertion indeed*; and declares, that if such experiments were lawful, and consistent with the rules of Christian practice, *he wishes to God it were also true.*

He maintains that inoculation is *no security against future infection*; and *that this practice contributes no more to the preservation of mankind, than it would contribute to the safety of a fortified town, to open the gates, invite the enemy*

*enemy, and surrender, lest, at some future time, the place should be taken by surprise.*

Dr. Deering, of Nottingham, another opponent of variolous inooculation, in the year 1737, published a pamphlet, in which he related an instance of the natural small-pox occurring after inoculation. The subject of it was a child of Dr. Croft; inoculated by Dr. Steigarthal, Physician to King George the First. Dr. Deering was an eye-witness of the operation; and assures us, *great care was taken in the choice of matter.* He had the small-pox of the *confluent kind, and in a severe manner;* and yet had it again *very full, in the natural way,* twelve months after. This, says Dr. Woodville, in his History of Inoculation, p. 217, *is a striking fact, which has never been contradicted.*

Dr. Pierce Dod, Fellow of the College of Physicians, and Physician to St. Bartholomew's Hospital, also opposed inoculation on the same ground; contending, that it affords no security against a future attack of the disorder. He published a case which occurred in a son of Mr. Richards, member for Bridport, who was inoculated for the small-pox. About sixty pustules came out; which maturated, scabbed, and went off in the usual manner. Two years afterwards he had the disease again, more severely, in the natural way. This case was communicated to Dr. Dod by Dr. Brodrepp, a learned and experienced physician, the grandfather of the child, who attended him on both occasions. Dr. Dod considers the doubts expressed by the friends of inoculation, concerning infection having taken place in that operation, as a mere excuse; as Dr. Rowley and Dr. Moseley have since considered the doubts expressed concerning vaccination.

Dr. Woodville, in his History of Inoculation, p. 134, says, "*one of the rumours spread, with a view to prejudice the public against inoculation, was, that this art seldom produced the genuine small-pox; and therefore would not secure the inoculated from the effects of variolous infection in the natural way.*"

Dr. Hilary, of Bath, in a Treatise on the Small-pox, published in the year 1735, considers the question, whether inoculation affords as great a security as the natural small-pox, as still undecided.

Dr. Maddox, Bishop of Worcester, the first President of the Small-pox Hospital, in a sermon preached for the benefit of that institution, observes, that in the infancy of the practice of inoculation, several objections had been urged against the practice, which time and experience had then removed. One of them was, *that the disease was more likely to return after inoculation.*

Dr. Mead, in his Treatise on the Small-pox and the Measles, published in 1747, alludes to the same objection, that inoculation is no security against a future attack of the small pox; and endeavours to refute it.

In Paris, Dr. Hecquet gave his opinion, that inoculation does not prevent the small-pox; and Dr. Woodville informs us, that although, in the year 1758, inoculation had extended into various parts of France, yet reports prevailed, that some persons, after inoculation, *had been seized with the natural small-pox.*

De Haen, in a tract published at Vienna, in 1757, affirms, that those who *undergo the inoculation of the small-pox, are not thereby secured from the future infection of the natural disorder.*

At Berlin, Dr. Baylies inoculated seventeen persons, in the year 1774; two of whom, a few weeks after, had *an eruptive disorder, of which one of them died.* This disorder was supposed, *by those who saw it,* to be the small-pox; but, *by those who did not see it,* to be a putrid fever. Certain it is, however, that it brought inoculation into general disiepute at Berlin.

In a small tract published at Boston, in New England, in the year 1722, which I received from Dr. Waterhouse, entitled, "Inoculation of the Small-pox, as practised at Boston," the author declares, *that those who were inoculated, often had the small-pox afterwards in the natural way.*

Mr. Moore, in his Reply to the Anti-Vaccinists, says, "the contest whether vaccination invariably prevents the small-pox, is exactly similar to that which was agitated a century ago, whether the inoculation of the small-pox invariably prevented the contracting of that disease afterwards by contagion, which continues an undecided point; for there are many cases fully authenticated, of persons having the small-pox twice; and many more have been narrated by the best authorities, which no one took the trouble to record. Dr. Woodville, late Physician to the Small-pox Hospital, often said, that patients were frequently brought into the hospital covered with the small-pox, who declared that they had been inoculated in their infancy; and had learnt from their mothers that they had had the disease."

It is stated in the same article in the Medical and Chirurgical Review, that Dr. Woodville declared he had never never seen an instance of small-pox occurring twice in the same person. I have been assured otherwise by several persons; particularly by Mr. Ridout, of Paternoster-row, and Mr. and Mrs. King, the parents of a child, whom Mr. Ridout attended with Dr. Woodville. That case I have already related in this Journal.

It is also insinuated in the same article, that other diseases are occasioned by the cow-pock as often, or more often, than by the small-pox. This is what no man can believe; and consequently, what no man ought to insinuate: but when parliament is preparing new honours for Dr. Jenner, it is natural that his *outrageous* opponent should make one more effort, to depreciate his merit, and to pluck the laurel from his brow.

In a subsequent part of the same article, in the Medical and Chirurgical Review, it is asserted, that the history of the vaccine affection is still imperfect; and that only a part of the subject has been as yet investigated. Dr. Walker, however, tells us, in the last number of this Journal, that the *simple* subject of *cow-pox*, as produced on the human species, is now, happily, pretty well understood, in every quarter of the globe.

I have read Mr. Marson's remarks in the last number of the Journal with attention; but cannot perceive any reason to alter the opinions which I have already expressed. I have noticed two cases, in which the subjects had each been inoculated for the cow-pock eight times, but to no purpose; yet, when they were vaccinated with fluid matter, taken at an early period, infection took place in them both, in both arms, the first time. A very considerable number of other cases, which have fallen under my observation, or been communicated to me by unquestionable authority, corroborate the opinion, that when infection fails, it is generally owing to a fault in the inoculator, or the matter.

I have the misfortune to differ from Mr. Marson in another point also. He thinks, that if a constitution is healthy, the mere cutting up of a vacine vesicle, or irritation of the arm, is not capable of producing the symptoms which I have described. The contrary, however, appeared to be the fact, in the Clapham case, and in the case which I related; as well as in several others. At the same time, I am ready to allow, that those who are guilty of one kind of malpractice, are commonly guilty of more; and that in in these instances, where the sores were denuded, nothing was applied to defend them for many days; till the inflammation had made a very considerable progress. Events of

 this this sort may frequently be expected, when mere compounders of drugs are permitted to practice surgery; and *blacksmiths* to dabble in vaccination.

———————— quod medicorum est

Promittant medici, *tractent fabrilia fabri.*

However objectionable Mr. Marson's observations on the foregoing points may be, what he says concerning tests and criterions is, in my humble opinion, still more so. He tells us, what he depends on, if he is certain the matter is genuine, is the pulsation of the inoculated part; that when he perceives this, he is certain it will proceed; and that he thinks very little of *the breakage* of the vesicle. Others have thought little, and *too little* of *the breakage* of the vesicle; and the consequence of their indifference to that circumstance is well known. Dr. Clarke's communication has set, or ought to set, this question to rest.

---

I have now before me a Circular Letter, the production of Dr. Cayley of Ripon, which, as well as many other documents, affords a melancholy proof, that vaccination is not so generally understood, as Dr. Walker imagines; and, indeed, were Dr. Walker's opinion well founded, that the genuine cow-pock contains two sorts of matter, capable of producing two sorts of diseases, that circumstance alone would be sufficient to evince, that the subject is not quite so *simple,* as some people *sometimes* wish us to believe.

Dr. Cayley, after observing, that it is desirable to ascertain the general opinion of the faculty concerning vaccination, the success of each practitioner, the failures which have occurred, and the most effectual mode of promoting its progress; says, " A serious inquiry being *upon the point to be instituted* by government, *to ascertain those points,* the questions to be answered by medical gentlemen, I hope you will not take it amiss, that I transmit to you the following questions and regulations; requesting you to favour me with as early an answer as convenient."

The questions proposed by Dr. Cayley appear to me to be unobjectionable: not so the regulations. I therefore deem it a duty incumbent on me, and on other members of the medical profession who entertain the same sentiments ments with me, to express a dissent from the doctrines laid down by Dr. Cayley.

After recommending to the medical practitioners residing at Ripon, and at the different towns within the distance of twelve miles, to form themselves into an association, for the laudable purpose of medical improvement; and particularly that of promoting vaccination; he proceeds to point out the particular means by which, he conceives, this is to be accomplished.

First, he recommends to the members of the faculty within that district, *totally to abandon the inoculation of the small-pox*; and observes, that the majority are ready to enter into a written engagement to this effect, provided their neighbours will do the same, and not encroach on their practice. This is highly honourable in those gentlemen; and an example worthy of imitation.

He then recommends general vaccination twice a year, gratuitous or otherwise, as circumstances may admit; and where practicable, that of infants a month after their birth. This is unobjectionable. He then speaks of glass, which, on different accounts, is a less convenient receptacle for virus, than the ivory point, or vaccinator, which I long ago proposed; and which has met with general approbation. When the matter is to be used within a day or two, a lancet is preferable. But if a patient can be procured with the vesicle in a proper state, it is still more adviseable to transfer the fluid matter from arm to arm.

Dr. Cayley either has not seen the cautions given by Dr. Jenner and other eminent inoculators, concerning vaccination, or thinks them unworthy of regard. He directs the virus on glass *to be moistened with the steam of hot water*, or with a very small portion of *warm water*. This, or at least the first, is considered by many practitioners, to be a frequent cause of failure in vaccination.

He directs the lancet to be introduced horizontally under the cuticle, about *a fifth of an inch*; being careful *not to allow any blood to flow*. This is a hard task; and one which I apprehend, few practitioners will be able to fulfil. It is true he directs the operator to press upon the point of the lancet with the finger; to elevate and depress it; and, at length, to withdraw it from under that pressure. Many practitioners would think even this operation more severe than is necessary; but Dr. Cayley informs us, that he has often seen it performed in such a careless and slovenly manner, that several uneven scratches have been made; in consequence of which, sometimes the matter is washed away; away; and at other times, extensive inflammation is produced.

He advises his colleagues never to vaccinate any subject labouring under *any recent cutaneous affection* to a violent degree; and yet expresses his opinion, that it would be *a good thing, to inoculate children at periods when the measles and the hooping cough prevail.* This to me appears rather inconsistent. He says, "it has been stated upon good authority, that such as have taken *either the above diseases,* whilst under the influence of cow-pox, particularly the measles, *they* have had them in a much milder form." This is contrary to my observation; I have known the measles, when supervening, during vaccination, prove fatal in more instances than one; and as to the hooping-cough, I am perfectly convinced, that it is as often aggravated as alleviated by vaccination. Dr. Cayley himself is of opinion, that the former part of the foregoing regulation should not be infringed with regard to the measles; and that no one should be vaccinated when the eruption of that disorder appears.

He thinks *great nicety* is required in the taking of matter. He directs it to be taken from the eighth to the *eleventh* day. This, in the opinion of most inoculators, is rather too great a latitude; especially when it is considered, that what is properly called the eleventh day, that is the eleventh *inclusively,* is by a very great proportion of medical practitioners still considered the tenth. Such practitioners would therefore, in reality, take it on the twelfth. The best rule in this respect is, I apprehend, *the golden rule* of Dr. Jenner; not to take matter after the areola begins to spread.

When I suggest, that the latitude allowed by Dr. Cayley for taking matter is too great, I rather allude to the late period at which he allows it to be taken, than to the length of time which he specifies. It may generally be taken at an earlier period than what he mentions, and with advantage.

He says, that it should always be taken in a *limpid* state; and that if it is *purulent,* it will produce a spurious disease. Those who are conversant with the practice, will therefore be surprised at the directions which follow. He says, "the point of a lancet should be introduced with great caution, not the tenth of an inch, horizontally into the edge of *the scab covering the pustule,* in about four different parts of its circumference." He says, "the punctures tures should not communicate with each other, for that would remove *the scab,* which should be avoided if possible; and great care should be used not to penetrate through *the sac* into the subjacent parts." By such an accident, he says, "there would unavoidably be a mixture of other than vaccine matter."

He tells us, that the lancet must not be put up till the matter is almost dry: and that it "*will be fit for use for some time.*" This indefinite expression may lead to ill consequences. Some may interpret it to mean a week, others a month, and others a year. It is, however, well known, that a lancet charged with the vaccine virus rusts much sooner than one that is charged with small-pox matter; and that nothing has brought a more just censure on the practice than this method of preserving, or rather of contaminating matter.

It is also difficult to conceive, how *a pustule,* covered by *a scab,* should yield *limpid* virus; nor is it easy to understand what Dr. Cayley means by the *sac,* which he so seriously cautions us not to penetrate. It is, indeed, rather mysterious, how we are to elicit the contents of the *sac* without penetrating it.

These strictures appear to me the more necessary, on account of Dr. Cayley's being situated, as he tells us, in the centre of a district; and having, on many occasions, been favoured with the confidence of the medical practitioners in that part of the kingdom. Were an insulated practitioner to hazard such crude notions in print, we might pass them by with indifference, and suffer them to roll down the stream of oblivion; but when a great medical luminary, placed in the centre of a populous district, sets himself up as an oracle, and establishes a kind of solar and planetary system, it is a duty, though a painful duty, to point out the defects of that system. Those who compose that system may, indeed, flatter themselves that they have light; but if the light that is in them be darkness, how great is that darkness.

Mr. Marson seems to be surprised at my supposing, tha there are respectable practitioners who are not conversant with vaccine inoculation. Every day, however, convinces me more and more of the truth of that opinion; and I should as soon believe Dr. Walker, if he told me he had discovered the use of *the spleen,* as when he declares, that vaccination is now pretty well understood in every quarter of the globe.

Mr.

Mr. Marson's paper is written in a very candid style, and deserves further attention. He is of opinion, that if the matter is *good,* no particular address is required in the operation. My experience and observation continually prove the fallacy of this opinion. The matter may be *good,* but in *a very small quantity*; in which case, it is the more likely that no part of it may be lodged in the puncture. It may be too much diluted, or decomposed by heat, at the time when it is used, in consequence of being dissolved in warm water, or held over steam; or it may be inserted dry, and withdrawn too soon. The best mode of using matter that has been preserved, is to insert it dry; and to leave it some time in the puncture that it may dissolve; but it is so hard of solution, that *it frequently remains on the instrument, when sent back to be charged again.*

Matter, however, is not always good. It is sometimes taken at too late a period; and when glass is employed as a recipient, the vesicle is the sooner drained, and the latter charges will be the weaker. There is more difference between the charges of matter furnished by different practitioners, than Mr. Marson seems to be aware of. I sent twelve vaccinators to Mr. Woodford of Taunton, *of which only one failed.* He afterwards received twelve from a practitioner, who boasts of the number of charges he sends out, *of which only one succeeded.* If the young woman mentioned by Mr. Southams, was, in general, inoculated with virus *from the same quarter,* we have a right to conclude, that the inoculator had but two chances of success, or three at the most.

According to Mr. Marson's mode of reasoning, whether inoculation fails to produce infection, or an immoderate inflammation of the arm takes place, the fault, in general, is not in the practitioner but in the patient. This is a very convenient, and a very comfortable opinion for medical men; but one to which neither the public, nor the most experienced inoculators, as far as I can learn, in general assent.

Mr. Marson's remarks concerning the cutting up of a vaccine vesicle, appear to me rather obscure and unintelligible. He says, the *case* which I mentioned, *leans* to his proposal. He says, if the constitution of the patient had been healthy, the *mere cutting up* of a vaccine vesicle, or irritation of the arm, would not have produced the symptoms which I speak of; and yet the mere cutting up of the the pustule, in the Clapham case, appeared to be the cause of the child's death, although *he was before healthy*.

From the general tenor of Mr. Marson's letter, I apprehend he did not intend to declare positively, that the mangling of a vesicle, and the irritation of the arm, *could* not in a healthy patient produce so much mischief; but only to state it as a matter of opinion. I have, however, better authority for affirming, that although the patient was healthy, this operation did produce it. He trusts it will be allowed, and I readily allow, that there is such a thing as a bad habit; but he farther contends for what I can never allow, and never will allow, till stronger arguments are adduced, that the habit may be so much improved by regimen or medicine, that we should have little to fear from the cutting up of a vaccine vesicle, or the irritation of the arm. Nature admits of some latitude; but there are certain bounds, which no prudent practitioner will ever transgress.

Mr. Marson flatters himself, that Dr. Willan will support him in his argument. He seems, however, to know but little of Dr. Willan's opinion. Dr. Willan, it is true, admits that a disaster may now and then happen, even in this mild process; but he will never attempt to prove, that the vaccine vesicle may be destroyed, and the arm irritated, without hazarding the life of the patient.

Mr. Marson says, Dr. Willan's observations imply, that it would be well to attend to the constitution of our patients, previous to, or at the time of inoculation. In this respect, most people will agree with him; but unfortunately for his other opinion, that the injury done to the arm could not produce so much mischief as that which I have described, if the child had been healthy, a melancholy proof of the contrary occurs in the same Journal.

It is related by Mr. Smart, of Union Street, Bishopsgate Street; who tells us, the subject of it was *a very healthy child*, the matter good, and the progress of the vesicle regular till the sixth day; when he discovered that it had been violently squeezed and broken down. Mr. Smart has not mentioned how far the inflammation had proceeded when Saturnine applications were first used; but, as far as can be conjectured from his account, it was not till it had made a considerable progress. He says, *the arm* was *first wrapped* in cloths wet with aq. lith. com.; and two or three poultices made with it and crumb of bread, were applied over the chest and belly. He then tells us, that finding the cuticle very suddenly peel off, he he discontinued these applications. I have seen one case, in which extensive inflammation came on, where no injury whatever had been done to the arm. The child was teething, the sleeve tight, and the weather very warm. In this case, I dissolved two drachms of cerussa acetata in a quart of water, and continued the whole night with the child; applying a large compress every two or three minutes; and not venturing to leave that task to any one of the family to perform.

I cannot help feeling some degree of surprise, when I read, that although the vesicle had suffered so severe an injury on the sixth day, instead of applying any thing of a saturnine or cooling kind, Mr. Smart charged four lancets from one side of it. It is true, he cautioned the gentleman for whom he charged them, not to depend upon the matter; but the gentleman was not prudent enough to be deterred from using it by this admonition; in consequence of which, another child had nearly fallen a victim to his temerity. Such is "*the simple subject of the cow-pox; now,*" according to Dr. Walker, "*happily, pretty well understood in every quarter of the globe.*"

I agree with the Editors of this Journal in opinion, that the injury done to the vesicle was the cause of the subsequent mischief. It is, however, desirable to know whether the most efficacious means were used at the first appearance of that mischief.

Mr. Marson tells us, that although so much is said about *tests,* the test which he depends on, when he is certain his matter is genuine, is *the pulsation of the inoculated part.* From this remark it is probable, that Mr. Marson mistakes the meaning in which the word test is commonly used. He says, when he perceives this, he is *certain it will proceed.* The use of a test, however, in the opinion of some of the advocates for the practice, is not superseded by that circumstance; for it is then employed by some gentlemen, in order to ascertain how far the constitution is affected. But it is more necessary, when a vesicle is much injured, and prevented from passing through its regular course.

Mr. Marson's criterion appears to me full as uncertain as that proposed by Dr. Walker. Pulsation is no more a characteristic of the cow-pock, than of any other inflammatory affection. It was observed in the Clapham case, when the *pustule* was destroyed; and where, it is supposed, no cow-pock had ever existed; and also in another case, case, which in some measure resembled it, both in its origin and event.

Three children were inoculated with matter preserved on glass; in consequence of which one of them had a spurious pustule, which was broken in a few days. In another, the operation failed; but in the third, a healthy boy five years of age, the most serious and melancholy effect ensued. According to the description given by the mother, a pulsation commenced in the part inoculated soon after the operation. In this case, therefore, Mr. Marson would have been certain of success: in this expectation, however, he would have been woefully disappointed. Instead of a slow progress, and a mild train of symptoms, such as are rather consolatory than terrifying to the most timorous mother, this pulsation was followed by the most violent inflammation, which rapidly spread over the whole arm. and a considerable part of the chest. In four or five days from the time of Inoculation, a mortification took place on the arm, two inches in diameter; and the child died. It is said, that the stomach was found, on dissection, to be in some degree inflamed; which is not improbable, when the extent of the external disease is considered. My opinion of this case is, that the child was not inoculated with vaccine matter alone; but with a mixture of matter and blood, which *one* inoculator professes to think an innocent practice. If, however, it is innocent in other cases, it cannot be so when kept till the blood has arrived at a state of putrefaction; which, under certain circumstances, will soon take place. In this case, no cow-pock rose; which is a sufficient proof, that we must not trust to pulsations.

Dr. Cayley tells us, his reason for proposing his plan is, that he may be able to ensure a constant succession of cow-pock matter, and supply those practitioners who subscribe to it. He tells us, he has sometimes been "*promised with a couple of glasses,*" and disappointed. Possibly his disappointment may have been no great loss; at least, if glasses are charged with such venom as that above described.

---

The following information, which I have just received from a correspondent, will, I doubt not, prove highly gratifying to your readers. "You will be pleased at hearing, that a short time since, an account of another embassy, similar in its views to that set on foot by his Catholic lic Majesty, reached me from Russia. This second philanthropic expedition was set on foot by the Court of Petersburgh; and when my intelligence of its progress arrived, had propagated vaccination through the vast territories of Siberia and Tartary; and was about to enter the northern boundary of China. There is another country in Europe, which might long since have atchieved such glorious enterprises as Spain and Russia, had it listened to my suggestions."

To this important document I beg leave to add another; shewing that there are persons in our own country, who hold this discovery in due esteem.

"COW-POX INOCULATION.

"The following facts are laid before the public, for the encouragement of those, who entertain any doubt respecting the efficacy and success of Vaccine Inoculation; and to confirm others in their good opinion of that inestimable blessing.

"In March 1800, having previously informed myself of the safety and efficacy of the cow-pock, I began to inoculate my two parishes, Leckhamstead and Akeley, near Buckingham. I was induced to do this at that particular time, because the Grand Junction Canal was in its progress to my immediate neighbourhood; which, like other great works, employiag vast bodies of men from distant quarters, would introduce the small-pox. It was my wish, that the labourers of these parishes should have the benefit of the high wages given on such occasions, without being exposed to the dangers of that dreadful pestilence.

"Having been in the habit of administering medicines to the poor, my offer to inoculate them was very generally accepted; especially as most of these people are employed in milking. The common answer of such persons to my proposal was, '*we all know that nobody ever died of the cow-pock; and we all know that nobody ever had the small-pox after it.* But what an odd thing it is, that any body should ever think of inoculating with it.' For my part, I thought it very odd, these two facts being so generally known, that no one should have attempted it sooner.

"I had no intention of proceeding in this practice beyond my own parishes; but I was soon applied to by a clergyman, to whom I have been more than twenty years curate, to inoculate at Greens-Norton, near Towcester, the the small-pox having broken out there in two families. I readily consented, on condition that he would prepare the minds of the people to whom I was but little known. In this he met with opposition; and in the result, about five hundred persons were inoculated with the small-pox; and twenty-eight by me with the cow-pock.

"I started on the same day as the hired inoculator. On the eighth day I inspected the parties; and finding that they were all decidedly infected with the cow-pock, I desired them to give all the assistance they could to the people who were sickening very fast with the small-pox, and in great distress for nurses; two hundred at one time being in a helpless condition. Many of the twenty-eight patients vaccinated by me slept with the patients labouring under the small-pox; and even with some of those who died in a most dreadful state.

"The inhabitants of the neighbouring villages were satisfied with this test; and in the following month I vaccinated *more than a thousand persons;* who were apprehensive that a very great fair at Towcester, on old May-day, would spread the small-pox over the whole surrounding country. On the application of clergymen, and other respectable inhabitants, I have inoculated with the cow-pock, within ten miles of my residence, upwards of four thousand seven hundred persons; many of them in situations greatly exposed to infection.

"In the autumn of 1804, the small-pox raging among the people employed at the tunnel of the Grand Junction Canal, I vaccinated in the neighbouring towns of Stoke Bruen, Shuttlehanger, and Paulerspury, five hundred and seventy. In the summer of 1805, I inoculated two hundred and seventy at Potterspury, the small-pox being at that time in two houses of the village.

"In the whole of my practice I have inflexibly avoided accepting any fee or present, except in two instances, where I had no choice. I am therefore not to be treated otherwise than as an independent evidence. In that character I make the following declarations.

First.—After a practice of more than six years, no instance has occurred of any one inoculated by me, being afterwards infected with the small-pox.

Secondly.—I never, during that period, have seen a single arm that required surgical assistance; or any other dressing, farther than a little oil, or milk and water.

Thirdly.—I never knew an instance of life being endangered, gered, or a taint left in the constitution, by the cow-pock; on the contrary, I can produce persons who date a period of health, unknown before, from the turn of the cow-pock; this disease having apparently a tendency to cleanse the constitution.

"If any candid person wishes to be more fully informed, let him devote a fortnight to the full investigation, on the spot. I promise him the use of my lists; and recommendations to proper persons, in every parish where I have set my foot. This is the only method which I propose, of suporting the above assertions; as local benefit to my neighbours, and not public fame or emolument, has been the object of

*Nov.* 8, 1806. "S. T. A. REED,

Curate of Lechamstead and Akely."

This is one instance, in addition to a thousand others which might be adduced, proving that the prejudices of the people are not unsurmountable. Having pursued the same plan as Mr. Reed, that of inoculating the poor gratuitously at their own houses, from the time when vaccination was introduced, till the Royal Jennerian Society was established, and in many instances since that time, I am authorized to state, from my own experience, that there is no material obstacle to retard the practice, but the falsehoods which are circulated with so much industry by artful and designing men.

I am, &c.

JOHN RING.

*New-street, Hanover-square.*

